# Correction: MELK is an oncogenic kinase essential for mitotic progression in basal-like breast cancer cells

**DOI:** 10.7554/eLife.36414

**Published:** 2018-03-12

**Authors:** Yubao Wang, Young-Mi Li, Lukas Baitsch, Alan Huang, Yi Xiang, Haoxuan Tong, Ana Lako, Thanh Von, Christine Choi, Elgene Lim, Junxia Min, Li Li, Frank Stegmeier, Robert Schlegel, Michael J Eck, Nathanael S Gray, Timothy J Mitchison, Jean J Zhao

Wang Y, Lee Y-M, Baitsch L, Huang A, Xiang Y, Tong H, Lako A, Von T, Choi C, Lim E, Min J, Li L, Stegmeier F, Schlegel R, Eck MJ, Gray NS, Mitchison TJ, Zhao JJ. 2014. MELK is an oncogenic kinase essential for mitotic progression in basal-like breast cancer cells. *eLife* **3**:e01763. doi: 10.7554/eLife.01763.

Published 20, May 2014

In 2014, we reported the role of MELK as an oncogenic kinase (Wang et al., 2014), and our key findings were independently confirmed in the context of basal-like breast cancer (Touré et al., 2016) and melanoma (Janostiak et al., 2017). However, subsequent studies by Dr. Sheltzer’s group at CSHL (Lin et al., 2017; Giuliano et al., 2018), and also from our own laboratories (Huang et al., 2017), have yielded observations that are in conflict with our initial findings. While we stand by our original conclusion regarding the role of MELK in cancer cell proliferation, we recognize that these more recent findings raise important questions regarding the merits of MELK as a target for cancer drug development. Here, we summarize new observations that may reconcile some, though not all, of the discrepancies between Wang et al. (2014) and the subsequent publications. In particular, our recent work shows that cellular dependency on MELK is highly conditional upon cell growth conditions, and points towards the existence of distinct sub-populations that are intrinsically MELK-independent or are able to adapt to MELK depletion.

## Proliferation assays for MELK dependency

The conflicting sets of studies differed in multiple aspects of their experimental protocol. However, using our own reagents and reagents from Dr. Sheltzer’s group at CSHL, we were able to substantially reproduce key findings from these studies, under their respective experimental conditions. Specifically, we found that MELK is necessary for the proliferation of MDA-MB-231 cells under clonogenic conditions (Figure 1A, right upper panels), as reported by Wang et al. (2014) and Touré et al., 2016. However, MELK is indeed dispensable under higher density culture conditions (Figure 1A, right lower panels), consistent with findings by Lin et al. (2017) and Huang et al. (2017). Therefore, at least under 2D culture conditions, it appears that cellular dependency on MELK is highly dependent upon growth density.

**Figure 1.**
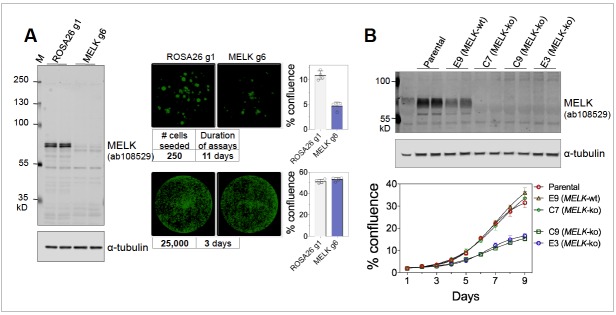
(**A**) Proliferation of MDA-MB-231, subjected to sgROSA26 (g1) or sgMELK (g6). Cas9-expressing MDA-MB-231 cells were seeded into 24-well plates at 250 or 25,000 cells per well, and imaged 11 or 3 days after seeding, respectively (Celigo Image Cytometry, Nexcelom Biosciences). Whole well images are shown with green pseudo coloring to visualize cell confluence. Graphs indicate mean ± SD (n = 4). Cas9-expressing MDA-MB-231 and lentiGuide constructs were generous gifts of Dr. Sheltzer. (**B**) Proliferation of MDA-MB-468 wild-type parental cells, MELK wild-type (E9) and MELK-knockout (E3, C7, and C9) clonal sub-lines under clonogenic growth conditions. Anti-MELK immunoblotting was visualized using the Odyssey CLx infrared imaging system (LI-COR Biosciences).

## MELK function in parental cell lines and clonal sub-lines

Our initial studies used inducible MELK knockdown in cell populations from non-clonal parental cell lines (Wang et al., 2014), in contrast to MELK-null clonal derivatives (Lin et al., 2017; Giuliano et al., 2018). We recently derived a number of MELK CRISPR knockout clonal sub-lines, and observed notable variation in proliferative capacity with some MELK-null clones proliferating at comparable rate to parental cells and others much slower (Figure 1B). Given that MELK may be essential for clonogenic growth as described above, clonal sub-lines are likely enriched for sub-populations that are MELK-independent or have adapted to its depletion.

## Off-target effects of genetic and chemical reagents

Our original studies were primarily based on RNAi-mediated MELK depletion experiments, whereas newer studies utilized CRISPR-Cas9 technology. In hindsight, we must acknowledge that these shRNA reagents, which were state-of-the-art at the time, have substantial off-target effects, and that these off-target effects may have contributed to their anti-proliferative effects. However, we note that the effects of MELK-targeting shRNAs can be rescued by re-expression of shRNA-resistant MELK (Wang et al., 2014; Janostiak et al., 2017), indicating that on-target MELK depletion contributed, at least in part, to the anti-proliferative effects observed. We also found that compounds targeting MELK (OTSSP167, HTH-01-091), like all chemical inhibitors, have a spectrum of other targets (Huang et al., 2017). This brings inevitable complexities to studies utilizing MELK inhibitors, and likely explains the persistence of their anti-proliferative effects upon MELK depletion.
